# Data transmission delay compensation algorithm for interactive communication network of offshore oil field operation scene in bad weather

**DOI:** 10.1371/journal.pone.0317137

**Published:** 2025-01-06

**Authors:** Jing Xiang, Xin Du

**Affiliations:** Cnooc Information Technology Co., Ltd., Shenzhen, Guangdong, China; Whale Wave Technology Inc, CHINA

## Abstract

A data transmission delay compensation algorithm for an interactive communication network of an offshore oil field operation scene in severe weather is proposed. To solve the problem of unstable microwave signals and a large amount of noise in the communication network caused by bad weather, the communication network signal denoising method based on Lagrange multiplier symplectic singular value mode decomposition is adopted, and the communication network data denoising process is realized through five steps; phase space reconstruction, symplectic geometric similarity transformation, grouping, diagonal averaging, and adaptive reconstruction. Simultaneously, the weak communication signal is compensated after being captured, that is, the characteristics of the weak signal are enhanced. On this basis, the communication network signals are collected to obtain delay information, and the data transmission delay of the communication network is predicted using the neural network algorithm based on long-term and short-term memory; Based on the prediction result of transmission delay, the PID controller is used to calculate the transmission delay compensation amount of the communication network, and the calculation result of the compensation amount is input into the delay compensator to realize transmission delay compensation. The experimental results show that the algorithm can effectively improve the quality of data transmission in the communication network and has a good delay compensation effect to ensure real-time communication.

## 1. Introduction

In offshore oil field operations, bad weather conditions have always been an important factor that affects the efficiency and safety of operations [1]. Offshore oil fields are usually located in remote sea areas, and extreme climatic conditions, such as typhoons, huge waves, and sea fog, not only increase the complexity of the working environment but also greatly challenge the stability of the communication system and real-time data transmission [2]. In this context, it is important to construct an efficient and reliable communication network to ensure the timeliness and accuracy of data transmission to ensure the safety and efficiency of offshore oil field operations [3]. With the progress in science and technology, communication technology is moving towards higher speeds, wider coverage, and lower delays. Data transmission delays are a problem that cannot be ignored in offshore oilfield operations. Time delay not only affects the real-time performance of data but also causes security risks, such as misoperation and equipment failure [4]. Therefore, it is of great significance to study the data transmission delay compensation algorithm of interactive communication networks in offshore oil field operation scenarios to improve operational efficiency and ensure operational safety.

Offshore oil field operation is a highly complex engineering activity, which is usually carried out in the sea far away from land and involves huge equipment and manpower inputs [5]. In such an environment, an efficient communication network is crucial to ensure the real-time transmission and processing of data, thus supporting the smooth progress of field operations. In an offshore oil field operation scene in severe weather (such as typhoons, storms, lightning activities, sea ice, etc.), the stability and reliability of the communication network are facing huge challenges owing to the complex marine environment and severe weather conditions [6]. Under severe weather conditions, the oil field will shake, leading to the instability of microwave signals, data transmission instability, or data interruption, which may seriously interfere with the normal operation of the communication network, resulting in data transmission delay [7] or even data loss, bringing many challenges to offshore oil field operations. When data encounters a delay during transmission, especially a long delay, it will not only affect the real-time performance of the data [8], but may also affect the performance and stability of the entire system. Therefore, developing an algorithm that can compensate for data transmission delays has important practical applications in such scenarios.

When Aleksandrov et al. studied the problem of communication delay compensation, they mainly focused on the impact of communication delay on communication control, built a first-order integrator and a double integrator, reduced the transmission delay, and improved the transmission efficiency by adaptively adjusting the packet transmission power, thus achieving communication delay compensation control [9]. In practical applications of this method, there is a high probability of serious packet loss during signal transmission. Sezgin et al. proposed a delay compensation strategy based on a decentralized real-time controller for the signal delay problem in the communication network and implemented a compensation design for the transmission delay by using an improved real-time computing packet queuing delay algorithm, thereby restoring the initial communication signal waveform [10]. The actual application of this method is significantly affected by the external environment, which leads to an unsatisfactory final delay compensation. Banerjee et al. designed an efficient CMOS active rectifier with adaptive delay compensation for the signal delay problem of a communication system and evaluated the optimal design conditions for achieving high compensation efficiency through mathematical analysis [11]. In the practical application of this method, the propagation signal contains considerable noise information. Qingsong studies the delay compensation of a neutral delay control system by cascade observers and proposes that the output feedback controller based on observers be designed to predict the future state, so that the input delay that can be arbitrarily large but accurately known can be completely compensated. Compared with prior art, for the stability of time-delay systems, simpler necessary and sufficient conditions are provided to ensure the stability of closed-loop systems. Finally, a lossless transmission line control system is designed to verify the effectiveness of the proposed controller [12]. However, the algorithm has received insufficient research on bad weather, and the effect of data transmission delay compensation is poor. Wang et al. studied the robust stability of networked control systems with transmission delay and analyzed their application in a 2-DOF laboratory helicopter. Using a new quadratic polynomial negative qualitative method, stability analysis and control synthesis of networked control systems with uncertainty and transmission delay are studied. First, by considering two independent parameters, a new negative qualitative method of quadratic polynomials is proposed. According to the proposed quadratic polynomial negative qualitative method and delay product, new layered stability and stabilization conditions are provided. Finally, an experiment on a two-degree-of-freedom laboratory helicopter system shows the effectiveness of the established conditions [13]. However, this method increases the time delay caused by the communication during transmission. Huang J et al. studied the singular value decomposition of flexible tensor and its application in multi-sensor signal fusion processing. In this study, an adaptive signal decomposition technique called the second tensor singular spectrum decomposition was proposed. This technology is very suitable for multi-sensor information fusion processing. Through dynamic simulation and experimental signal analysis, the effectiveness of the proposed technology has been thoroughly evaluated. The comparative analysis shows that this method is superior to the traditional method in multi-sensor signal fusion processing, feature extraction, early fault detection and maintaining the internal relationship between multi-sensor signal attributes. However, under this method, electromagnetic interference may lead to the degradation of multi-sensor signal transmission quality, thus affecting the accuracy and timeliness of data transmission [14]. Huang J and others studied the first kind of flexible tensor singular value decomposition: the innovation of multi-sensor data fusion processing, and proposed a novel tensor singular value decomposition method, called the first kind of flexible tensor singular value decomposition. The first kind of flexible tensor singular value decomposition method provides unique advantages, avoids relying on the so-called tensor "flattening" and has stable decomposition results, thus solving the limitations of the traditional tensor SVD based on n- mode product. Inspired by the advantages of the first kind of flexible tensor singular value decomposition method and the principle of iterative decomposition, the first kind of tensor singular spectral decomposition is designed. This innovative method is outstanding in the field of multi-channel data fusion processing. Finally, the validity of this method is verified by the dynamic simulation signals and experimental data collected from bearing test-bed and gearbox used in engineering field. The comparison results show that the proposed method is superior to some existing methods. However, this method can’t fully adapt to the problem that the data transmission demand changes at any time, which leads to the increase of data transmission delay or data loss [15]. Xu H et al. proposed a semi-supervised multi-sensor information fusion clipping graph embedding low-rank tensor learning machine with very low labeling rate. In this study, a new semi-supervised classifier based on low-rank tensor was developed, which can effectively alleviate the above difficulties and improve the diagnostic accuracy in engineering applications. Firstly, the multi-sensor and multi-channel vibration signals are transformed into a pixel matrix, which is superimposed as the feature tensor of multi-sensor information fusion to maintain the coupling relationship between multi-sensor signals and realize the reasonable fusion of multi-source features. In addition, an advanced tensor decomposition method, tensor kernel norm, is introduced into the GE-LRTLM model to obtain the low-rank structural information of each feature tensor, so as to extract the most important features and patterns from tensor data and keep the tensor data structure unchanged. Finally, manifold regularization and tensor-based graph construction methods are introduced to obtain potential labeled information from unlabeled samples, so as to better describe the geometric similarity and distribution of tensor data. A large number of semi-supervised experiments are carried out on multiple data sets, and the experimental results show that the classification accuracy of this method can reach 97% even if the number of labeled samples is extremely limited. At the same time, it is also verified that the combination of labeled and unlabeled multi-sensor information fusion tensor samples can promote the improvement of model accuracy. However, this method is affected by many factors, and the instability further aggravates the data transmission delay problem.

In view of the above problems, this paper studies the data transmission delay compensation algorithm of an interactive communication network in an offshore oil field operation scene in bad weather. Aiming at the large amount of noise information and significant delay in the interactive communication network data of offshore oilfield operation scenarios under adverse weather conditions, a compensation algorithm is proposed to achieve delay compensation. The innovation of this algorithm is as follows:

1. A communication network signal denoising method based on Lagrange symplectic multiplier singular value mode decomposition is adopted, which achieves communication network data denoising through five steps: phase-space reconstruction, symplectic geometric similarity transformation, grouping, diagonal averaging, and adaptive reconstruction.
2. Simultaneously the weak communication signal is compensated after being captured, that is, the characteristics of the weak signal are enhanced.
3. Collect communication network signals to obtain delay information and use neural network algorithms based on long short-term memory to predict the data transmission delay of the communication network;
4. Based on the prediction results of the transmission delay, a PID controller is used to calculate the transmission delay compensation amount of the communication network, and the calculation result of the compensation amount is input into the delay compensator to achieve transmission delay compensation.

## 2. Data transmission delay compensation algorithm for interactive communication network in offshore oilfield operation scenarios

### 2.1. Signal enhancement of interactive communication network

In the offshore oil field operation scenario during severe weather conditions (including typhoons, storms, lightning activity, sea ice, etc.), the oil field experiences vibrations, leading to instability in the microwave signal and the generation of significant noise. Consequently, prior to compensating for the data transmission delay in the interactive communication network within the offshore oil-field operation scenario, it is imperative to enhance the communication signal. This enhancement process aims to improve the accuracy of the data transmission delay compensation within the interactive communication network of offshore oil field operations. The enhancement process comprises two key components: communication network signal denoising based on Lagrange multiplier symplectic singular value decomposition, and weak communication signal amplification processing.

#### 2.1.1. Communication network signal denoising based on Lagrange multiplier symplectic singular value mode decomposition

The communication network signal denoising method utilizing Lagrange multiplier symplectic singular value decomposition can be succinctly outlined in five steps: phase-space reconstruction, symplectic geometric similarity transformation, grouping, diagonal averaging, and adaptive reconstruction.

*2*.*1*.*1*.*1*. *Phase space reconstruction*. The given interactive communication network signal of a discrete offshore oilfield operation scenario is expressed as *x* = {*x*_1_,*x*_2_,⋯,*x*_*n*_}, *n* refers to the length of the interactive communication network signal in the offshore oil field operation scenario. According to Takens’ Embedding Theorem, multidimensional interactive communication network signals from offshore oil field operation scenarios can be reconstructed from one-dimensional signals. In other words, all original time series can be utilized to construct the signal trajectory matrix *X* for the interactive communication network of offshore oil field operation scenarios.


$$X=[\begin{array}{ccc} x_{1} & \cdots & x_{1+(d-1)\tau }\\ \vdots & \cdots & \vdots \\ x_{m} & \cdots & x_{m+(d-1)\tau } \end{array}]$$ (1)

Where, *d* represents an embedded dimension, *τ* represents the delay time, window lengthd *m* = *n*−(*d*−1)*τ*. Generally,The value of *τ* is 1, which can be calculated through the power spectral density *d*.

*2*.*1*.*1*.*2*. *Symplectic geometric decomposition*. Covariance matrix of signal trace matrix of interactive communication network based on offshore oilfield operation scenario *A* = *X*^*T*^*X* construct Hamilton matrix *M*:

$$M=[\begin{array}{l} A\;0\\ 0\;-A^{T} \end{array}]$$ (2)

According to the theorem and definition of symplectic geometry, the construction of symplectic orthogonal matrix Q requires another Hamilton matrix *N* = *M*^2^eparture, i.e.:

$$Q^{T}NQ=[\begin{array}{l} H\;R\\ 0\;H^{T} \end{array}]$$ (3)

Where, *Q* protect the structure of Hamilton matrix during transformation. here, *H* represents the upper triangular matrix, that is *h*_*ij*_ = 0(*i*>*j*+1).The matrix can be orthogonalized by Schmidt *N* transform, the characteristic value of upper triangular matrix *H* can be calculated as *λ*_1_,*λ*_2_,⋯,*λ*_*d*_. In fact, if *A* is real symmetric, then the characteristic value of *A* will be equal to the characteristic value of *H*. According to the properties of Hamilton matrix *A* characteristic value of *σ*_*i*_ expressed as:

$$\sigma_{i}=\sqrt{\lambda_{i}}(i=1,2,\cdots ,d)$$ (4)

Distribution of *σ*_*i*_ represents of Symplectic geometric spectrum of *A*, *Q*_*i*_(*i* = 1,2,⋯,*d*) represents the eigenvector corresponding to the eigenvalue of matrix *A*. The characteristic values of *A* are arranged in descending order, namely:

$$\sigma_{1}>\sigma_{2}>\cdots >\sigma_{d}$$ (5)

*2*.*1*.*1*.*3*. *Grouping*. According to Formula (5), in *σ*_*i*_ the smaller value is usually regarded as the noise component, so it is very important to select the singular value of the signal reconstruction of the interactive communication network in an offshore oil-field operation scene. The second order modified contribution rate method is used to select the effective singular values.

According to $\sigma_{i}^{\prime }=\sqrt{\sigma_{i}^{2}-\sigma_{d}^{2}}(i=1,2,\cdots ,d)$, the contribution rate of each singular value is calculated respectively after the singular value sequence is modified:

$$\eta_{1}=\frac{\sigma_{i}^{\prime }}{\sum_{i=1}^{d}\sigma_{i}^{\prime }}\times 100\%$$ (6)

When the contribution rate is not more than 50%, the corresponding *i* as the initial noise reduction order *k*_1_ and then calculate its cumulative contribution rate. Current contribution rate *η* when its value is greater than 95%, the corresponding *i* is chosen as the order of noise reduction *k*_0_. At this time, the noise power in the interactive communication network signal of offshore oilfield operation scene can be expressed by $a_{k}^{c}$.

Using the modified contribution rate method, you can choose the one that contains the main information *k*_0_ Symplectic geometric component. According to the properties of Hamilton matrix, the reconstructed trajectory matrix Z its calculation process is as follows:

$$\left\{ \begin{array}{l} \sigma_{i}=\sqrt{\lambda_{i}}\\ Z_{i}=Q_{i}Q_{i}^{T}X^{T}\\ Z=Z_{1},Z_{2},\cdots ,Z_{i}(i=1,\cdots ,k_{0}) \end{array}\right.$$ (7)

When the noise is strong, the symplectic geometric component of the interactive communication network signal of the offshore oil field operation scene retained by the second-order modified contribution rate method still contains some noise. Therefore, it is necessary to filter this noise. The time domain constraint estimation method is used to remove residual noise. Reconstructed trajectory matrix Z can be expressed as: *Z* = *PX*^*T*^, *P* transformation matrix representing connected symplectic matrix, connected symplectic matrix *J* = *A*^2^ can be regarded as linear superposition of an interactive communication network signal matrix of pure offshore oilfield operation scene *S* and noise matrix *N*. The error matrix of the interactive communication network signal and its estimation matrix in the pure offshore oilfield operation scene is expressed as *R*, we can get:

$$R=(P-I_{d})S+PN$$ (8)

The time domain constraint estimation formula of the error matrix is as follows:

$$\min{\left\|\left((P-I_{d})S\right)\right\|}_{F}^{2},s.t.{\left\|PN\right\|}_{F}^{2}\leq \alpha d\sigma_{w}^{2}$$ (9)

Where, ‖‖_*F*_ represents Frobenius norm, *α*≤[0,1] is a preset threshold to limit the residual noise of interactive communication network signals in offshore oil field operation scenes.

An optimization problem with inequality constraints can be solved by constructing a Lagrange function to obtain a local solution. The Lagrange function of the optimization problem expressed in Eq (10) is as follows:

$$L(P,v)={\left\|\left((P-I_{d})S\right)\right\|}_{F}^{2}+v({\left\|PN\right\|}_{F}^{2}-\alpha d\sigma_{w}^{2})$$ (10)

According to the Karush-Kuhn-Tucker condition, we can get:

$$P_{v}=U_{1}(I_{k}-\sigma_{w}^{2}\Lambda_{1}^{-2}){\left(I_{k}-(1-v)-\sigma_{w}^{2}\Lambda_{1}^{-2}\right)}^{-1}U_{1}^{T}$$ (11)

Where, *U*_1_, Λ_1_ is obtained from the connected symplectic matrix *J* block matrix form.

After filtering, the signal reconstruction matrix of interactive communication network in offshore oilfield operation scene can be expressed as:

$$Z=U_{1}F_{v}U_{1}^{T}X^{T}$$ (12)

As can be seen from Eq (12), the matrix *F*_*v*_ diagonal element $f_{v}(i)=\frac{1-\sigma_{w}^{2}/\sigma_{i}^{2}}{1-(1-v)\sigma_{w}^{2}/\sigma_{i}^{2}}$ filtering singular values *σ*_*i*_ has an important role in.

*2*.*1*.*1*.*4*. *Diagonal averaging*. To decompose the original time series into independent superimposed components with different trends and frequency bands, the original time series is analyzed. Z is the convenience of calculation, the parameters are defined as follows: For any initial one-component matrix, *Z*_*i*_, defining the elements of the matrix as *z*_*ij*_. Thus, D single-component signals are obtained by diagonal average:

$$Y=Y_{1},Y_{2},\cdots ,Y_{d}$$ (13)

*2*.*1*.*1*.*5*. *Adaptive reconfiguration*. Although these components are not entirely independent of each other, they share the same period and require the reconstruction of the initial single component with identical characteristics. Hence, it is imperative to analyze and reconstruct each component based on similarity, in order to obtain independent *SSC*_*s*_. The Pearson correlation coefficient is employed to quantify the similarity between two discrete series, and its formula is as follows:

$$\rho (A,B)=\frac{\sum_{i}^{N}\left(\frac{A_{i}-\mu_{A}}{\sigma_{A}})(\frac{B_{i}-\mu_{B}}{\sigma_{B}}\right)}{N-1}$$ (14)

Where, *μ* represents the average, *σ* represents the standard deviation. Set the threshold of correlation coefficient and carry out significance test. When *R*>0.95 and *P*<0.05, the two components are considered to be the same mode, and the two components are added. Owing to the interference from adverse weather and environmental factors, interactive communication network signals in offshore oil field operation scenarios often contain substantial noise components that lack discernible regularity in terms of frequency and correlation. Consequently, it is necessary to establish termination conditions for reconstruction iterations.

First, because the main components are distributed at the front of the matrix, the first initial single component is compared. *Y*_1_ and adding the similar components with high similarity to obtain the first reconstructed component. *SSC*_1_. Then from the matrix *Y* remove *SSC*_1_, the rest of the matrix can be expressed as *C*_1_. Finally, the residual signal is obtained by summing the residual signals *g*^1^. And then calculate the normalized mean square error between the residual signal and the original signal:

$$NMSE^{\Lambda }=\frac{\sum_{e=1}^{n}g^{h}(e)}{\sum_{e=1}^{n}x(e)}$$ (15)

Where, *h* represents the number of iterations. When the normalized mean square error is less than a given threshold, *th* = 1% i.e. The decomposition process will end. Otherwise, the residual matrix is used as the original matrix to repeat the iterative process until the iterative stop condition is met, and the final decomposition result is obtained:

$$x(n)=\sum_{h=1}^{N}SGC^{k}(n)+g^{\left(N+1\right)}(n)$$ (16)

Where, *N* represents the number of components, the residual energy will decrease at each iteration.

#### 2.1.2. Weak communication signal amplification processing

In bad weather, the compromised signal quality of the interactive communication network in offshore oil field operation scenarios is partly attributed to the presence of weak signals in addition to the aforementioned noise interference. The existence of these weak signals can result in the loss of communication information. Consequently, it is essential to amplify weak communication signals within the interactive communication network signals of offshore oil-field operation scenarios. This process primarily comprises two key components: capturing weak communication signals and compensating for them within the interactive communication network signals of offshore oil-field operations.

*(1) Weak communication signal capture part*. The weak communication signal capture component of the interactive communication network signal in offshore oil-field operation scenarios employs an improved differential coherent accumulation algorithm that incorporates half-bit and circular shift operations. This enables the capture of weak communication signals through the application of Fourier transform.

(2) After the weak communication signal is captured, it enters the compensation part, that is, it enhances the characteristics of the weak signal. The specific process is as follows:

Step 1: Establishing the power amplifier;

Step 2: Perform a predictive test and establish a transfer function based on test results;

Step 3: Mapping the input communication signal of the offshore oil field operation scene interactive communication network and the output communication signal individually by using the transfer function to obtain the nonlinear characteristics of the signal transmission channel of the offshore oil field operation scene interactive communication network;

Step 4: Establish a lookup table based on the obtained nonlinear characteristics;

Step 5: Obtain the compensated original offshore oil field operation scene interactive communication network signal waveform by mapping the relationship between the input offshore oil field operation scene interactive communication network communication signal and the output communication signal. This process involves signal acquisition, preprocessing, design, and implementation of the compensation algorithm and an in-depth technical analysis of the compensated signal waveform. The core of the compensation algorithm is to identify and correct various distortions caused by the channel such as attenuation, noise, and multipath effects. Once the compensation algorithm is successfully applied to the output signal, the signal waveform can be significantly improved and becomes closer to the stationarity and characteristics of the original signal. This improvement is not only reflected in the stability of the signal amplitude, but also in the signal integrity, signal-to-noise ratio, spectrum characteristics, and accuracy of the phase information. A comprehensive survey of the signal waveform, from the signal fluctuation range and spectrum distribution to phase continuity, is conducted in detail. Through these analyses, the performance of the compensation algorithm is quantified, and the necessary optimization is carried out accordingly to ensure the reliable transmission of signals in the interactive communication network of offshore oil field operation sites. Finally, when the compensated signal waveform reaches or approaches the expected quality standard, it can be applied to an actual environment to support efficient and safe operation of offshore oil fields.

### 2.2. Design of delay compensation algorithm

#### 2.2.1. Communication network data transmission delay prediction based on Neural network algorithm for long and short memory

The prediction of data transmission delay in communication networks based on long-term memory neural networks is an advanced method used to predict the delay time of data transmission in communication networks. This method uses the long-term memory ability of LSTM to effectively capture the long-term dependence in time-series data to accurately predict network delay. The LSTM neural network algorithm is an improved version of the recursive neural network, which is particularly suitable for processing time-series data. In the data transmission delay prediction of the communication network, the LSTM neural network can learn the patterns in the historical delay data and predict future delays based on these patterns. By training a large amount of historical data, the LSTM model can learn the potential laws in the data to accurately predict of future delays. With time, the data transmission delay of the offshore oil-field operation scene interactive communication network gradually accumulates, resulting in an increasingly serious delay. To compensate for the next delay, it is necessary to clarify the transmission delay of the offshore oil field operation scene interactive communication network signal at the current moment. Therefore, after enhancing the communication network signal of the offshore oil field operation scene in bad weather, the communication network signal is collected to obtain delay information, and a neural network algorithm based on long-short memory is used to predict the data transmission delay of interactive communication networks in offshore oil field operation sites.

The LSTM neural network algorithm is an improved version of a recursive neural network, and its topology is a chain network composed of several identical neurons connected sequentially. The basic structure of neurons contains three functional structures: the forgetting gate, input gate, and output gate. Use *X*_*t*_ to represent the time series data input of neurons, that is, to enhance the transmission delay of interactive communication network signals in offshore oil field operation scenes; *h*_*t*_ is output the time sequence data of neurons at time t; *S*_*t*_ is the output state memory unit, which is used to transmit the transmission delay information of historical communication network signals.

When neurons are connected in chain in time sequence, the input of neurons at time t includes (*t*−1) time neuron output *h*_*t*−1_ and output that data of the state memory unit *S*_*t*−1_ and *X*_*t*_. The outputs of neuron forgetting gate, input gate and output gate are as follows:

$$\left\{ \begin{array}{l} S_{t-1,f}=S_{t-1}\times \sigma (w_{f1}h_{t-1}+w_{f2}X_{t}+b_{f})\\ S_{t-1,i}=\sigma (w_{i1}h_{t-1}+w_{i2}X_{t}+b_{i})\times \tan h(w_{i3}h_{t-1}+w_{id}X_{t}+b_{i2})\\ h_{t}=\sigma (w_{o1}h_{t-1}+w_{o2}X_{t}+b_{o})\times \tan h(w_{o3}S_{t}+b_{o2}) \end{array}\right.$$ (17)

Where: *σ* represents the Sigmoid function, and its output range is [0,1]; *w* represents the weight of the corresponding function; *b* represents the offset of the corresponding function; In the subscript, *t* represents the moment, *f* represents forgetting gate, *o* represents the output gate.

A Sigmoid function is used to control the degree of forgetting information. Output 1 indicates that all the transmission delay information of the communication network signals is circulated, 0 indicates that all the transmission delay information of the communication network signals is forgotten, and between 0 and 1 indicates that the transmission delay information of the communication network signals is partially forgotten. In this way, long- and short-term neural networks can realize the transmission and forgetting of transmission delay information of communication network signals. In a single neuron, the parameters of Sigmoid function of the forgetting gate, input gate, and output gate control the forgetting degree of the transmission delay information of the communication network signals between these gates. Long-term and short-term neural networks realize an information memory path between time-series signals through the chain connection of multiple neurons [16]. At time t, three continuous time sequence signals are transmitted *X*_*t*−2_, *X*_*t*−1_ and *X*_*t*_ is delivered to the input layer of chain connection in turn, and output from the output layer after passing through the hidden layer of chain connection *t*+1 time forecast data *X*_*t*+1_.

#### 2.2.2. Communication network transmission delay compensation

**Fig 1:**
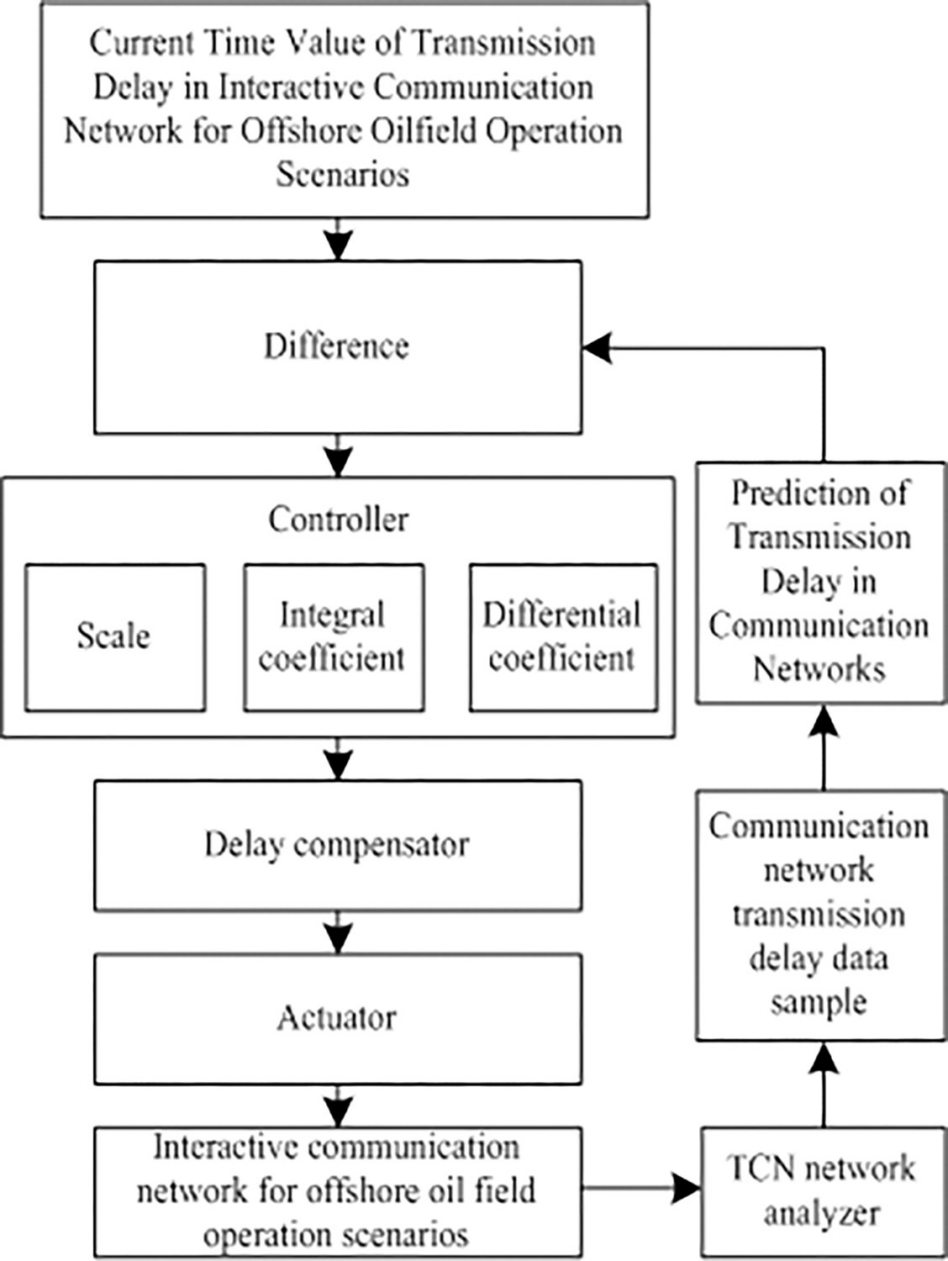
Communication network transmission delay compensation.

Based on the prediction results of the communication network transmission delay, combined with a PID controller, the data transmission delay compensation of the interactive communication network in an offshore oil field operation scene in bad weather is realized [17], and the specific process is shown in Fig 1.

The specific process of communication network transmission delay compensation is as follows:

1. Inputting communication network transmission delay information based on long- and short-term neural network prediction;
2. In the process of communication network transmission delay compensation, obtaining the reference value of delay Δ*H* is a continuous process that comprehensively considers network monitoring data, historical analysis, model prediction, current network environment and transmission conditions. Including collecting network performance indicators by real-time monitoring system, revealing the law of delay change through historical data analysis, establishing a prediction model based on statistics or machine learning algorithm to predict future delay, and fully considering the influence of network topology, routing, transmission medium, equipment performance and environmental factors (such as bad weather). Finally, according to this information, a reference value of delay Δ*H* is set, which is both in line with the actual situation and has a certain safety margin. This value will be applied in the actual network environment and continuously optimized to ensure the real-time and reliability of network transmission. Calculate the delay difference Δ*H* between the current time and the predicted time by Formula (18):

ΔH=‖H(t)−H(t+1)‖ (18)

Where, *H*(*t*) represents the communication network transmission delay value at time t, *H*(*t*+1) represents the communication network transmission delay value at time t+1, that is, the predicted communication network transmission delay value at the next moment.

(3) Enter Δ*H* into the value PID control section, three sub-controllers are used to calculate the transmission delay compensation of communication network [18], and the formula is described as follows:

$$\left\{ \begin{array}{l} G_{1}(t)=K_{p}\Delta H\\ G_{2}(t)=\frac{1}{T_{i}}\int \Delta Hdt\\ G_{3}(t)=T_{d}\frac{d\Delta H}{dt} \end{array}\right.$$ (19)

Thus, the overall compensation amount is obtained, and the calculation formula is as follows:

$$y(t)=G_{1}(t)+G_{2}(t)+G_{3}(t)$$ (20)

Where, *y*(*t*) represents the delay compensation at time t, *G*_1_(*t*), *G*_2_(*t*), *G*_3_(*t*) represents time t *K*_*p*_, *K*_*i*_, *K*_*d*_ output of three sub-controllers, *T*_*i*_ represents the integration time constant, *T*_*d*_ represents a differential time constant.

(4) The calculation result of the compensation amount is input into the delay compensator [19] to realize the delay compensation of data transmission in the interactive communication network of an offshore oil field operation scene in bad weather. The delay compensator model is described as follows:

F(t)=ψ(k⋅y(t)+Δt∫L) (21)

Where, *F*(*t*) represents a delay compensator model, *ψ* represents the delay compensation coefficient [20], *k* represents the signal attenuation factor of the interactive communication network of the offshore oil field operation scene in bad weather, Δ*t* is transmission interval of interactive communication network representing offshore oilfield operation scene, *L* indicates the length of the communication network packet transmission queue.

Through the above process, delay compensation of data transmission in the interactive communication network of an offshore oil field operation scene in bad weather can be realized.

## 3. Experimental results

In this study, the data transmission delay compensation algorithm of an offshore oil field operation scene interactive communication network in bad weather is studied. To verify the practical application performance of this algorithm, an offshore oil field operation scene interactive communication network in a far sea area is selected as the research object. The research object includes six oil-field operations, with only three main communication microwave networks leading to land, which are far away and have a small bandwidth. In the entire oil field, a set of databases is formed by collecting all wireless microwave and other meteorological data, providing three data synchronization servers (mainly used for the distribution of all routes at sea to ensure the smooth data of each oil field at sea), which are placed on three main platforms. The three links corresponding to the three data synchronization servers have a bandwidth of approximately 1000Mbps at the highest signal and are interrupted at the lowest signal, with an average bandwidth of approximately 800Mbps and a distance of about 150km. Microwave transmission is used as a backup between oil production platforms, and each oil field provides satellite communication for emergency use. By judging the real-time signal, bandwidth, and flow in the database, the data of each platform at sea can be automatically planned. The intelligent transmission of offshore oilfield data should be realized to ensure the stable operation of offshore oilfield communications. The data transmitted include three types of data: internal network of oil field (voice, office email, office network), private network (confidential data of oil fields) and external network (daily online). Severe weather in the research object area is mainly typhoon weather and a gale of magnitude 12 or above.

### 3.1. Standing wave detection results

**Fig 2:**
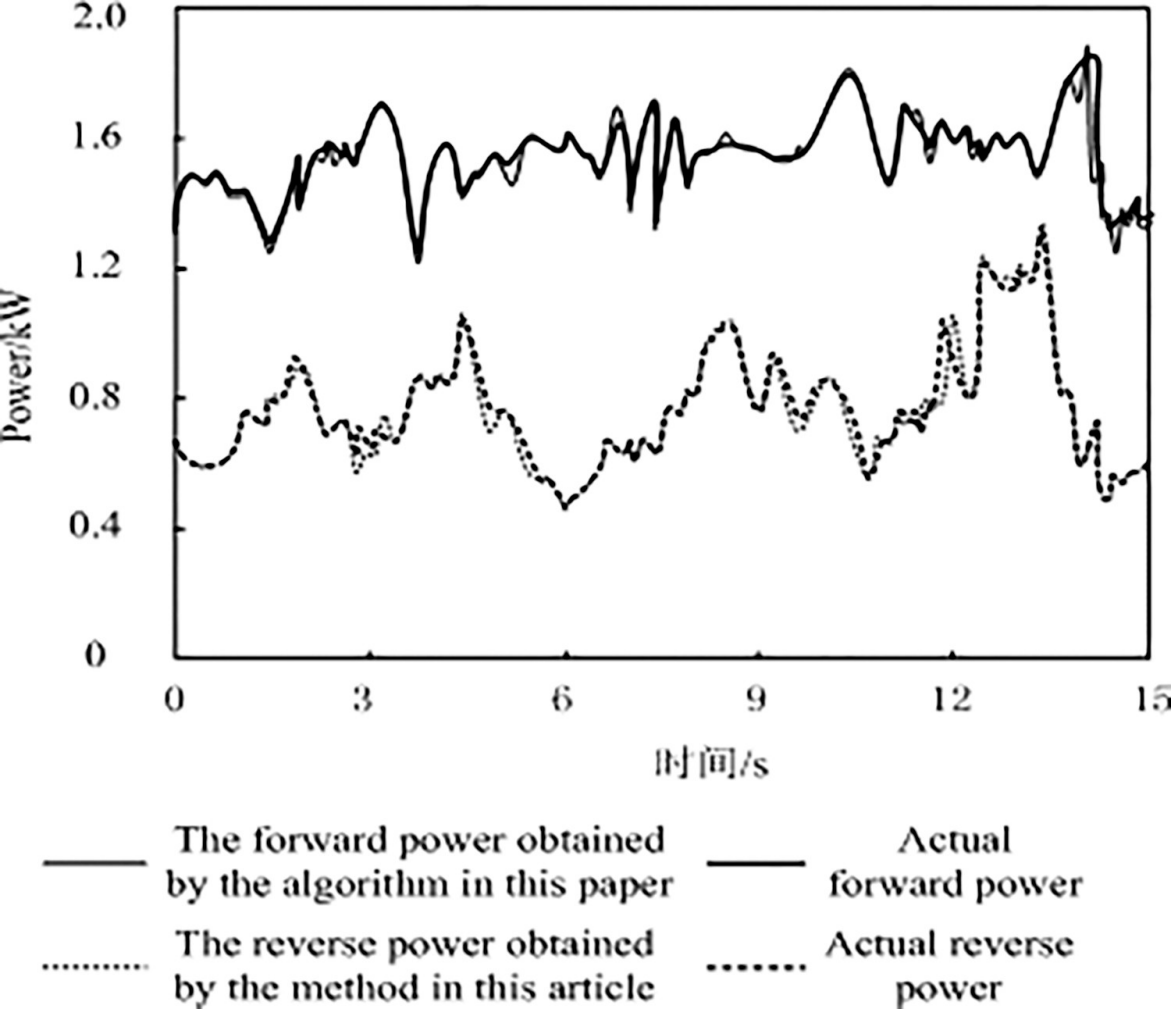
Standing wave test results.

The comparison of the standing wave acquisition results of the communication signal source of the research object obtained by this algorithm with the actual standing wave situation of the research object is shown in Fig 2.

Through an in-depth analysis of Fig 2, we can clearly see that when the algorithm measures the forward and reverse powers of the communication signal source of the research object the results obtained are very close to the actual standing wave power of the research object and almost overlap. This discovery not only proves the accuracy of the algorithm in data acquisition but also reveals its powerful ability to deal with the standing wave power of communication signal sources. The algorithm accurately calculates and analyzes the forward and reverse powers of the communication signal source to obtain measurement results that are highly consistent with the actual standing-wave power. This is due to the outstanding performance of the algorithm in data processing, signal analysis, and error control. It can not only accurately capture every subtle change in the signal source output but also effectively filter out interference and noise to ensure the accuracy and reliability of the measurement results.

### 3.2. Communication signal noise suppression

**Fig 3:**
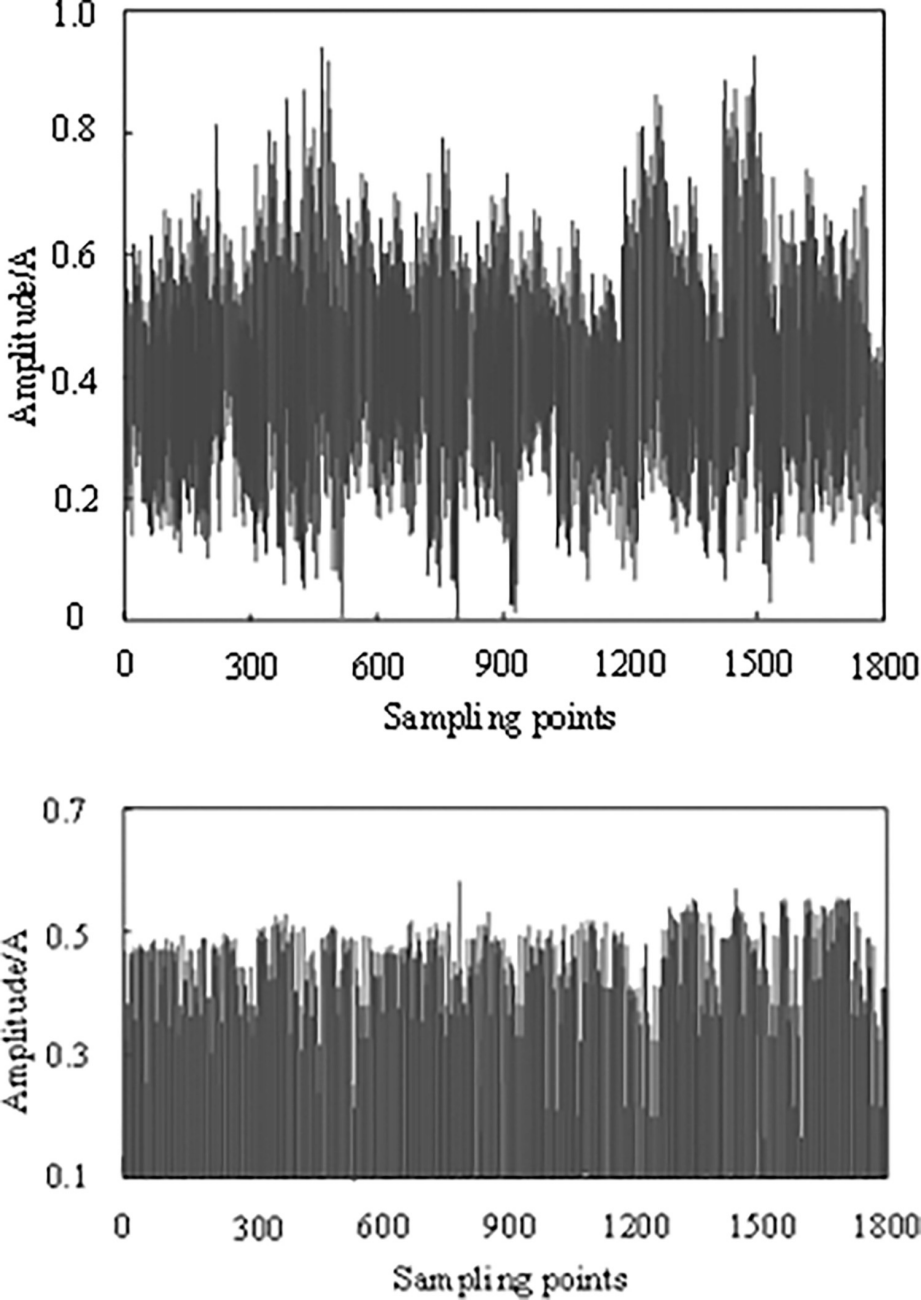
The denoising performance of the algorithm in this article. (a) Before denoising, (b) After denoising.

Fig 3 shows the signal noise filtering performance of the algorithm in this study under bad weather conditions.

As shown in Fig 3(A), the communication signals collected by the research object are periodically weakened in a bad weather environment. This weakening not only affects the transmission quality of signals but also seriously threatens the security and stability of communication information. Owing to the signal attenuation and distortion caused by weather factors, it is difficult to accurately extract and identify the effective information in the original signal, thereby increasing the uncertainty and risk in the communication process. As shown in Fig 3(B), by applying this algorithm, the communication signal is effectively enhanced, which not only completely suppresses the noise components in the signal but also better preserves the useful information in the collected signal. This improvement makes the signal more stable during the transmission process and improves the reliability and efficiency of the communication.

Specifically, the algorithm analyzes and optimizes the original signal through a series of complicated calculations and process. It can not only accurately identify the noise components in the signal but also intelligently enhance it according to the characteristics of the signal, so that the quality of the signal is significantly improved. At the same time, the algorithm also has a high adaptive ability, which can be adjusted and optimized in real time according to the signal changes in different weather environments to ensure that the communication process is always in the best state.

### 3.3. Weak signal compensation test

**Fig 4:**
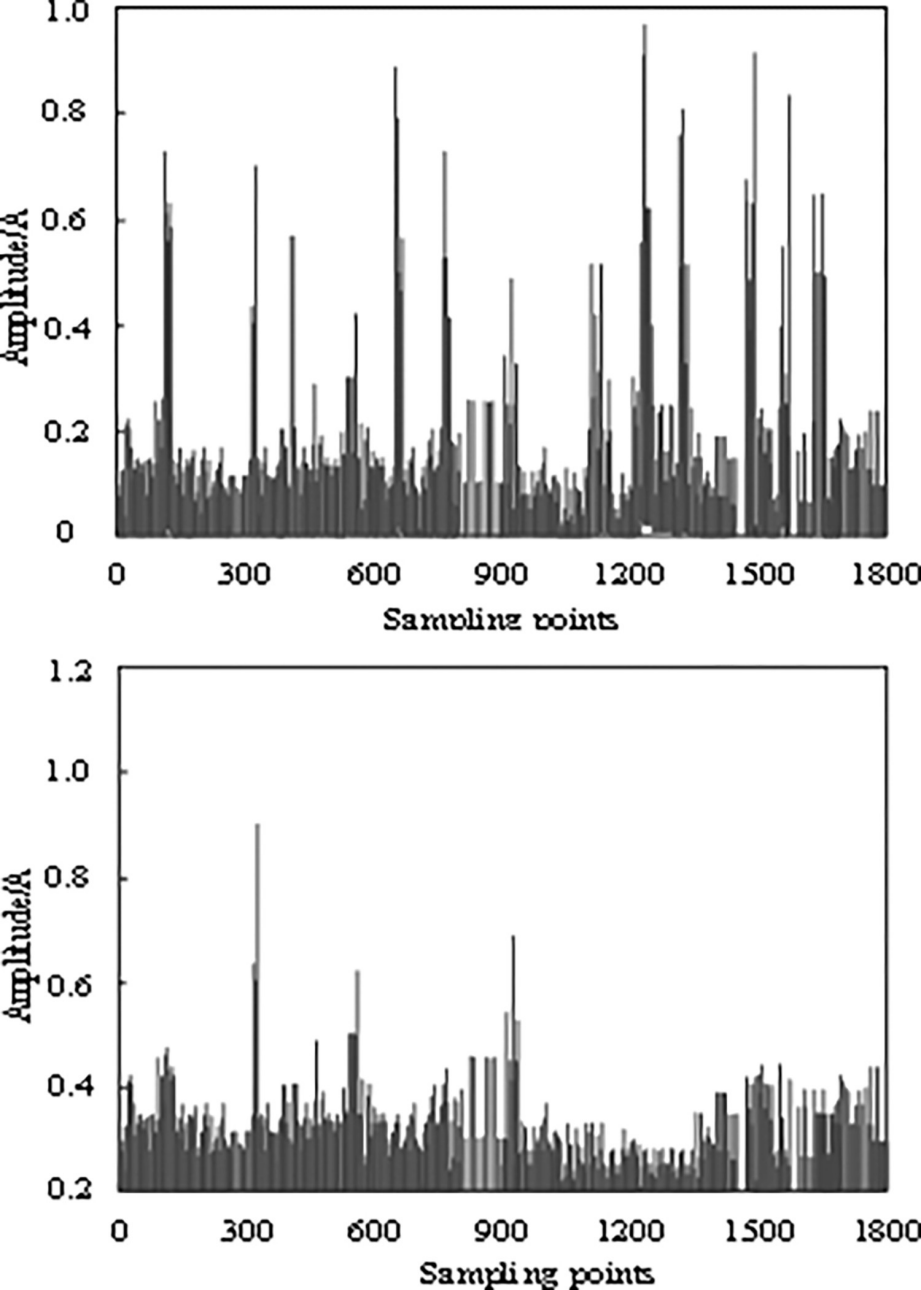
Weak signal compensation results. (a) Before compensation. (b) After compensation.

Fig 4 shows the compensation results for weak signals in the communication process of the research object before and after adopting the algorithm in this study.

By analyzing Fig 4 and using this algorithm to amplify and compensate for the weak signal in the communication process of the research object, the effect is remarkable. After such processing, the residual fluctuation of the communication signals at different sampling points is significantly reduced, which is almost negligible. This discovery not only proves the effectiveness of the algorithm in weak signal processing but also highlights its outstanding ability to improve the quality of communication signals.

### 3.4. Delay prediction performance

**Fig 5:**
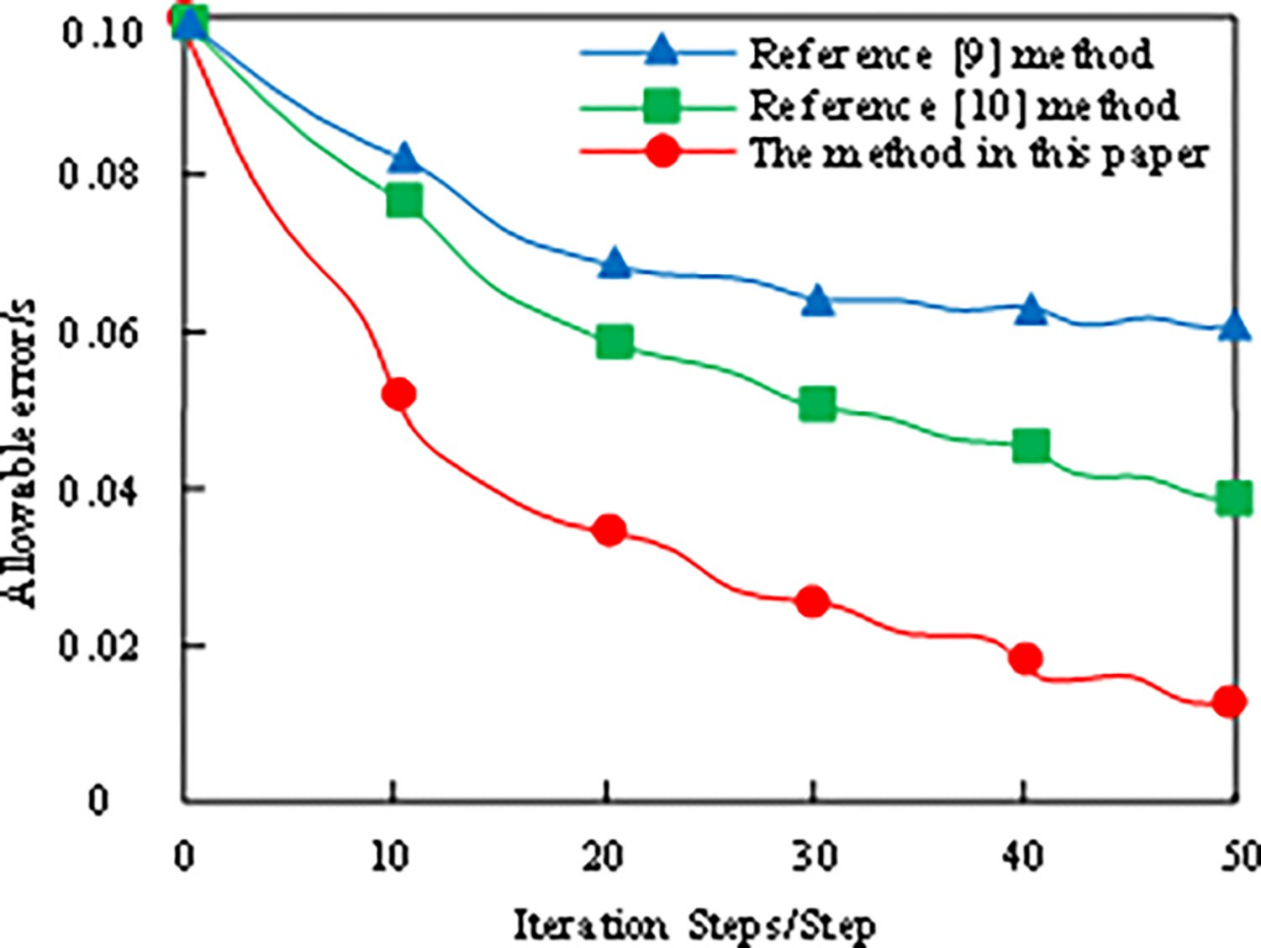
Comparison of training results of different methods of delay compensation prediction model.

Delay prediction refers to the process of predicting a possible delay in data transmission according to historical data, current network status, and possible future influencing factors in the network communication system. This prediction identifies and compensates for the transmission delay caused by network congestion, signal attenuation, transmission distance increase, or specific environmental factors in advance, thereby ensuring the real-time reliability of data transmission. In this paper, the initial parameters of long-term and short-term memory neural network algorithm are set as follows: the structural parameters of long-term and short-term memory neural network are 4/36/1; Maximum permissible error is 0.02; The learning rate is 0.25; The connection of each layer is 0.14 and 0.3/0; The connection threshold of each layer is 0.036/0.44; The maximum number of iterations is 100, the delay time is of the order of 0.1 s or even shorter (10 ms). Under bad weather conditions, the operating environment of offshore oil fields frequently changes. A shorter delay time enables the system to respond to these changes more quickly and adjust the operation strategy and parameters to cope with unexpected situations and challenges. Under the aforementioned initial parameter settings, taking the method of reference [9] and the method of reference [10] as the comparison method, and the method of this paper as the experimental method, the delay prediction model based on long-term and short-term neural networks is trained by using three methods. The comparison result is shown in Fig 5.

Through the analysis of Fig 5, it can be seen that the delay prediction errors of the methods in Reference [9] and Reference [10] are relatively high. When the iteration steps are 40, the delay prediction error of the method in this paper is less than 0.02, and the training process of this method has been successfully completed, so it is feasible in the actual delay compensation application.

### 3.5. Delay compensation results

**Fig 6:**
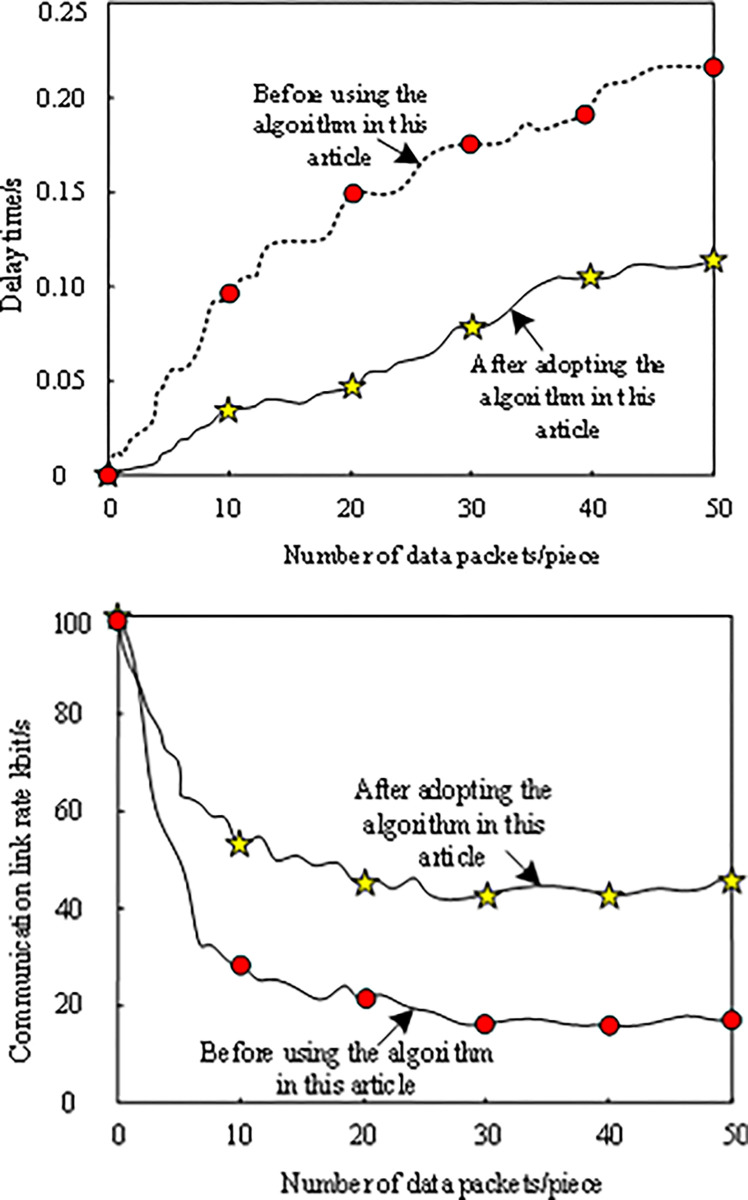
Communication delay compensation results. (a) Delayed. (b) Communication link rate.

Under the same test conditions, the communication delay of the research object is compensated by this algorithm, and the communication delay and communication link rate of the research object before and after this algorithm compensation are obtained. The results are shown in Fig 6.

From the analysis in Fig 6, after employing this algorithm for compensation, the communication delay of the research object has been notably shortened, and the communication link rate has also been significantly enhanced. This finding demonstrates the superior performance of the algorithm in terms of delay compensation. Not only does the algorithm optimize the communication delay, but it also boosts the communication link rate, thereby offering a robust guarantee of communication system stability and efficiency.

### 3.6. Communication delay compensation effect test

**Fig 7:**
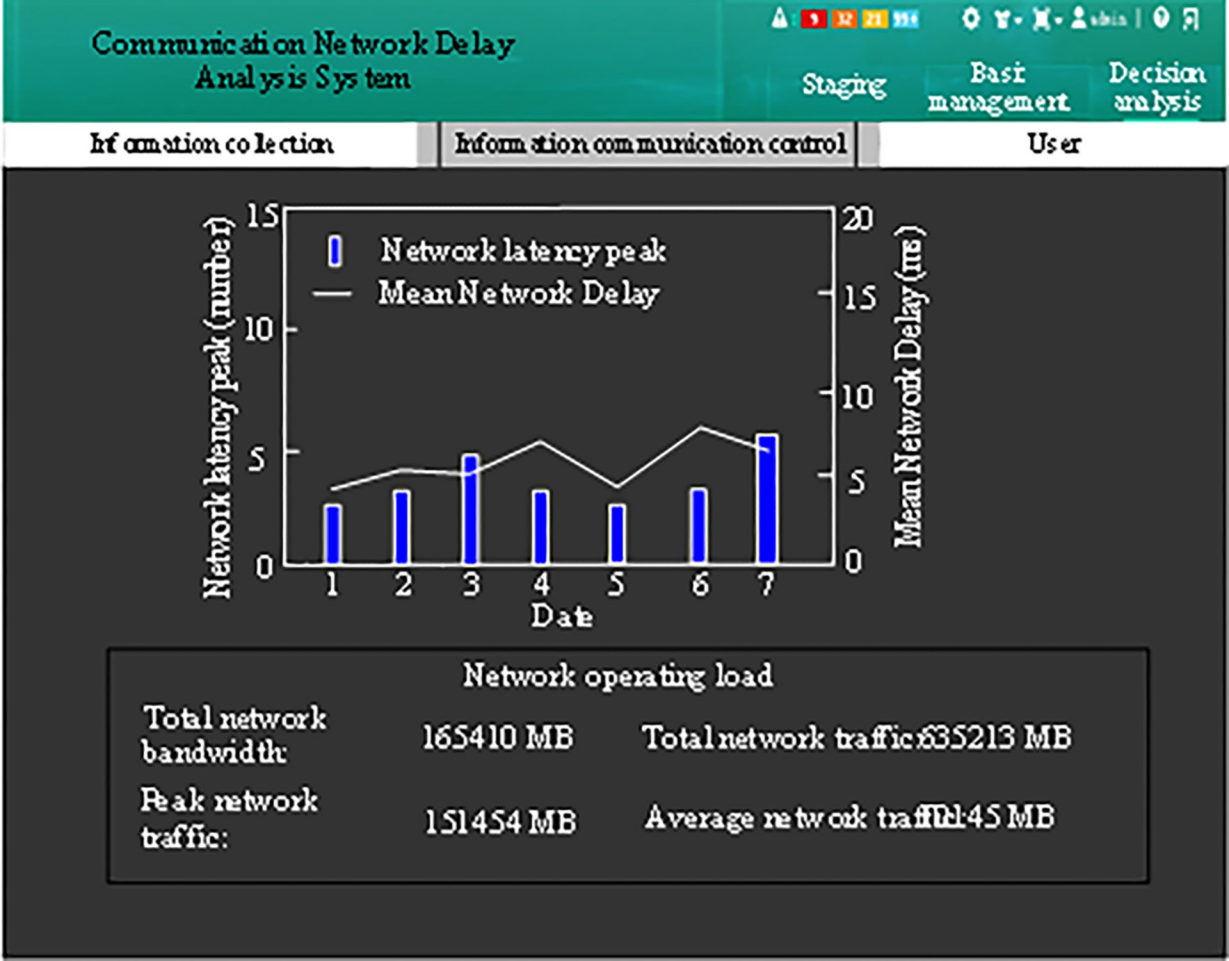
Communication network delay compensation results.

Test the results of communication network delay compensation from November 1 to November 7, 2022 after the application of the algorithm in this paper, as shown in Fig 7.

From the analysis of Fig 7, it can be observed that after the application of this algorithm, both the number of network delay peaks and the average value of network delay have decreased significantly, with the average value of network delay peaks being less than 5 and the average network delay being reduced to less than 10 ms. This clearly demonstrates that the algorithm presented in this paper can effectively ensure the real-time communication of the research object.

## 4. Conclusion

With the continuous improvement in informatization and automation of oilfield production in recent years, increasing attention has been paid to the interactive communication of offshore oilfield operation scenes. In this study, the data transmission delay compensation algorithm of an interactive communication network in an offshore oil field operation scene under bad weather conditions is studied. After improving the data transmission quality of the communication network, transmission delay compensation is realized. The experimental results verify the application performance of this algorithm in improving the quality of data transmission in communication networks and compensating for transmission delays. However, research on this algorithm still needs to be improved, that is, the delay packet loss problem is not taken into account in the process of transmission delay compensation, which leads to the packet loss problem in the practical application of this algorithm when the transmission data is large, and will be further expanded and further studied in the follow-up research process.
